# Context-dependent expression of a conditionally-inducible form of active Akt

**DOI:** 10.1371/journal.pone.0197899

**Published:** 2018-06-19

**Authors:** Soyeon Park, Robert E. Burke, Tatyana Kareva, Nikolai Kholodilov, Pascaline Aimé, Thomas F. Franke, Oren Levy, Lloyd A. Greene

**Affiliations:** 1 Department of Biological Sciences, Columbia University, New York, New York, United States of America; 2 Department of Pathology and Cell Biology, Columbia University Medical Center, New York, New York, United States of America; 3 Department of Neurology, Columbia University Medical Center, New York, New York, United States of America; 4 Department of Neuroscience, Icahn School of Medicine at Mt Sinai, New York, New York, United States of America; University of PECS Medical School, HUNGARY

## Abstract

Akt kinases are key signaling components in proliferation-competent and post-mitotic cells. Here, we sought to create a conditionally-inducible form of active Akt for both *in vitro* and *in vivo* applications. We fused a ligand-responsive Destabilizing Domain (DD) derived from *E*. *coli* dihydrofolate reductase to a constitutively active mutant form of Akt1, Akt(E40K). Prior work indicated that such fusion proteins may be stabilized and induced by a ligand, the antibiotic Trimethoprim (TMP). We observed dose-dependent, reversible induction of both total and phosphorylated/active DD-Akt(E40K) by TMP across several cellular backgrounds in culture, including neurons. Phosphorylation of FoxO4, an Akt substrate, was significantly elevated after DD-Akt(E40K) induction, indicating the induced protein was functionally active. The induced Akt(E40K) protected cells from apoptosis evoked by serum deprivation and was neuroprotective in two cellular models of Parkinson's disease (6-OHDA and MPP^+^ exposure). There was no significant protection without induction. We also evaluated Akt(E40K) induction by TMP in mouse substantia nigra and striatum after neuronal delivery via an AAV1 adeno-associated viral vector. While there was significant induction in striatum, there was no apparent induction in substantia nigra. To explore the possible basis for this difference, we examined DD-Akt(E40K) induction in cultured ventral midbrain neurons. Both dopaminergic and non-dopaminergic neurons in the cultures showed DD-Akt(E40K) induction after TMP treatment. However, basal DD-Akt(E40K) expression was 3-fold higher for dopaminergic neurons, resulting in a significantly lower induction by TMP in this population. Such findings suggest that dopaminergic neurons may be relatively inefficient in protein degradation, a property that could relate to their lack of apparent DD-Akt(E40K) induction *in vivo* and to their selective vulnerability in Parkinson's disease. In summary, we generated an inducible, biologically active form of Akt. The degree of inducibility appears to reflect cellular context that will inform the most appropriate applications for this and related reagents.

## Introduction

The serine/threonine kinase Akt, also known as protein kinase B (PKB), is a critical downstream effector of the PI3K signaling pathway [1–5]. Akt is comprised of three highly conserved domains: an N- terminal pleckstrin homology (PH) domain, a kinase domain with serine/threonine phosphotransfer specificity, and a hydrophobic C-terminal tail. Canonical Akt activation begins when a trophic factor binds to a receptor tyrosine kinase and activates PI3K, which phosphorylates lipids at the plasma membrane to generate phosphatidylinositol 3,4 bisphosphate (PI3,4P_2_) and phosphatidylinositol 3,4,5 triphosphate (PIP_3_). These phosphorylated lipids exhibit high affinity for the Akt PH domain and lead to its recruitment to the inner surface of the plasma membrane. Membrane translocation of Akt and binding of its PH domain to phospholipids leads to a conformational change from a “PH-in” to a “PH-out” conformation, allowing access of upstream kinases to the kinase domain and phosphorylation at two sites: Thr308 within the catalytic domain by PDK1 and Ser473 within the C-terminal hydrophobic domain by mammalian target of rapamycin complex 2 (mTORC2) and other kinases [6]. The degree to which the two canonical phosphorylation sites have distinct functions has not been fully determined; however, it has been reported that while phosphorylation of both sites is required for full catalytic activity, phosphorylation of the Thr308 site stimulates enzymatic activity by an estimated 100-fold, whereas phosphorylation of the Ser473 site can induce activity levels by an additional 7 to 10-fold [7–11].

Once Akt is in a catalytically active state, it can in turn interact with over 100 purported substrates to induce a variety of downstream physiological effects, including cell growth, proliferation, survival, and altered metabolism [1, 5]. While much of the focus on Akt's functions has been related to proliferation-competent cells, especially in cancer, important roles for Akt have been defined in post-mitotic cells such as neurons. This includes promotion of neuronal survival, differentiation and regeneration [12–14]. Abnormal Akt signaling has also been associated with neuropsychiatric and neurodevelopmental disorders as well as with neurodegenerative disorders including prion disease and Parkinson’s disease (PD) [12, 15–19]. In the case of PD, there is evidence for deficient Akt signaling in affected patient neurons [18, 19] and that over-expression of constitutively active Akt promotes neuroprotection and axonal regeneration in animal models of PD [20, 21].

Fully understanding the functional roles of Akt in cells and harnessing its activity for potential therapeutic applications has posed several challenges. Among these is that activation via growth factor signaling or elevation of PI3K activity involves regulation of additional pathways, while over-expression has the potential drawback that the timing and level of protein produced are difficult to control. Moreover, for possible therapeutic use, overexpressed Akt poses the potential hazards of unregulated activity and in view of finding increased Akt activity in a wide variety of human cancers, potential for promoting neoplasms. For these reasons, we sought to develop a tunable, inducible form of Akt for use both *in vitro* and *in vivo*. To achieve this, we drew on the Destabilizing Domain (DD) technology introduced by the laboratory of Dr. Thomas Wandless [22]. This approach involves the fusion of a destabilizing protein domain derived from *E*. *coli* dihydrofolate reductase (DHFR) to the protein of interest, conferring instability to the entire fusion protein via proteasomal degradation. Stabilization of the fusion protein is induced by the cell- and blood brain barrier-permeable, FDA approved antibiotic, Trimethoprim (TMP), which binds the DHFR domain and interferes with degradation in a concentration-dependent manner [22]. This system has been validated in a variety of biological contexts, including parasites [23, 24], mammalian cell lines [22, 25–27], and rodent brains [22, 26–28].

Here, we describe the creation of an inducible DD-Akt using the constitutively active Akt1(E40K) mutant. We have evaluated the system in cell culture and in brain and demonstrate that DD-Akt(E40K) is inducible and physiologically active in both proliferating cells and post-mitotic neurons. Intriguingly, our data suggest that the inducibility of DD-Akt(E40K) is dependent on neuron type and region of expression within the brain. Such findings raise potential limitations for use of DD-linked proteins in brain and may indicate differences in susceptibility of specific neuron types to pathological processes in neurodegenerative diseases.

## Materials and methods

### Cell culture

HEK293T/17 cells (purchased from the ATCC, catalogue number CRL-11268) were grown in DMEM supplemented with 10% FBS and penicillin/streptomycin. Cultures were transfected using Lipofectamine 2000 (ThermoFisher Scientific) according to the manufacturer's protocols. Trimethoprim (TMP; Sigma-Aldrich; 10 μM) was added to cultures as indicated 24 h after transfection, and protein extraction or immunofluorescence staining was performed 24 h after treatment.

PC12 cells (originally derived in our lab [28]) were cultured as described previously [29] on plastic cell culture dishes coated with rat tail collagen (Roche). Undifferentiated PC12 cells were grown in RPMI 1640 medium supplemented with 10% heat inactivated horse serum (Sigma), 5% FBS, and penicillin/streptomycin. For neuronal differentiation, cells were grown in RPMI 1640 medium supplemented with 1% horse serum, penicillin/streptomycin, and 50 ng/ml final concentration of human recombinant NGF (kind gift of Genentech and Gemini Bio-Products #300-174P). Lentivirus was added to the cultures at an approximate MOI of 5–10 on day 1–2 after plating for undifferentiated cultures and 4–5 days after NGF treatment for differentiated cultures. 10 μM TMP was added to cultures 4–5 days after infection, and protein extraction was performed 24 h after treatment.

Primary cortical cultures were prepared as previously described [30]. Briefly, cortices were isolated from E18 rat embryos, dissociated, and plated on poly-D-lysine (Sigma) coated plates at a density of 3 x 10^5^ cells/well. Cultures were maintained in Neurobasal medium (ThermoFisher Scientific) supplemented with 2% B-27 (ThermoFisher Scientific) and 0.5 mM glutamine (ThermoFisher Scientific). Half of the medium was changed every 3 days after plating. Lentivirus was added to the cultures at an approximate MOI of 5–10 on day 5–7 *in vitro* and protein extraction was performed 4–7 days after infection.

Ventral midbrain dopaminergic neurons from P0-P3 rats were dissected, dissociated, and plated on a confluent glial monolayer as described previously [31]. Briefly, 1 mm^3^ blocks were dissected from the substantia nigra of a coronal midbrain slice, according to anatomical landmarks, and enzymatically dissociated in a papain solution for 20–30 minutes. Digested tissue blocks were rinsed and triturated 10 times with large-bore tech tips, 10 times with medium-bore tech tips, and 10 times with small-bore tech tips. Cells were centrifuged, resuspended in SF1C medium, and plated at a density of 80,000 cells per dish on top of a glial monolayer. Cultures were treated with GDNF (10 ng/ml) and processed as needed for experiments. Lentivirus was added to the cultures at an approximate MOI of 10 on day 6–7 *in vitro*, 10 μM TMP was added to cultures 6–10 days after infection, immunofluorescence was performed after 4 days of TMP treatment.

### Cell death paradigms

#### Serum deprivation

For serum deprivation of undifferentiated PC12 cells infected with lentivirus expressing DD-Akt(E40K), cells were detached from wells with serum-free RPMI 1640 and pelleted at 1500 rpm for 5 min. Pellets were washed in serum-free RPMI 1640, repelleted, resuspended in serum-free RPMI 1640 and plated at a density of 5 x 10^5^ cells/ml in the presence and absence of either 50 ng/ml of NGF or 10 μM TMP. Cells were analyzed for survival 24 h after serum deprivation.

#### PD toxins

For neuronal PC12 cells, 10 mM stock solutions of 6-hydroxydopamine (Tocris) or 1-methyl-4-phenylpyridinium (MPP^+^) (Sigma) diluted in water were freshly prepared just before each experiment. 6-OHDA was used at final concentrations ranging from 100 to 150 μM, and MPP+ was used at a final concentration of 0.25 mM, for 16–24 h.

### Quantification of cell survival *in vitro*

For PC12 cells infected with lentiviral particles (typically achieving a 50% transduction rate in undifferentiated PC12 cells and 75% transduction rate in differentiated PC12 cells) and treated with PD toxins or deprived of serum, cell survival was assessed by counting the total cell population as previously described [32].

### Cloning

#### DD-Akt(E40K)

The DHFR-derived DD-YFP gene insert in a pBMN vector was obtained from Addgene (Plasmid #29325). The HA-Akt1(E40K) gene insert [33] from hereon in called Akt(E40K)) was amplified using PCR and primers as follows: Fwd 5' gcgcatgcTACCCATACGATGTTCCAGATT-3' and Rev 5'-atccgcggTCAGGCTGTGCCACTGG-3'. The amplified Akt(E40K) gene was inserted 3’ of the DD domain, replacing YFP, in a pBMN vector using restriction sites Sph1 and SacII to create pBMN-DD-Akt(E40K). The entire DD-Akt(E40K) gene was then inserted using restriction sites BamHI and SacII and blunt-end ligation into the overexpression vector pWPI (Addgene, Plasmid #12254), a bicistronic lentiviral vector allowing the simultaneous expression of the transgene and EGFP under the control of the EF1-α promoter.

#### DD-Akt(WT)

Akt(WT) was amplified from mouse cDNA using primers as follows: Fwd 5'-tctagcgcatgcATGAACGACGTAGCCATTGTGAA-3' and Rev 5'-cattatccgcggTCAGGCTGTGCCACTGGCTGA-3'. The amplicon was inserted 3’ of the DD domain, replacing YFP, in a pBMN vector using restriction sites Sph1 and SacII to create pBMN-DD-Akt(WT).

### Western immunoblotting

Cells were homogenized in 1x cell lysis buffer (Cell Signaling Technology, #9803) with LDS-sample buffer (Invitrogen) supplemented with 50 mM dithiothreitol and complete mini protease inhibitor mixture (Roche, #11836170001), or in 1x sample buffer (5x sample buffer: 250 mM Tris-HCl pH 6.8, 10% SDS, 0.5% bromophenol blue, 50% glycerol, 5% 2-hydroxy-1-ethanethiol). Lysates were boiled at 100°C for 10–20 min prior to analysis. A total of 20 μg of proteins was loaded per well of 10% or 4–12% Bis-Tris polyacrylamide gels (Invitrogen) and separated by electrophoresis for 1.5 h at 100 V. Proteins were then transferred onto a nitrocellulose membrane (Bio-Rad) for 1 h 30 min at 40 V. Membranes were blocked with 5% powdered milk in TBS containing 0.1% of Tween 20 (TBST) and incubated overnight at 4°C with primary antibodies.

The following primary antibodies were used for Western blotting: rabbit anti-phospho-Akt (pThr308) (Cell Signaling Technology #13038 [34], #2965 [35]), mouse anti-phospho-Akt (Thr308) (Cell Signaling Technology #5106 [36]), rabbit anti-phospho-Akt (Ser473) (Cell Signaling Technology #4060 [34]), rabbit anti-pan-Akt (Cell Signaling Technology #4685 [37]), rabbit anti-phospho-FoxO4 (pS193) (Cell Signaling Technology #9471 [38]), rabbit anti-total FoxO4 (Cell Signaling Technology #2499 [38]), mouse anti-actin (Sigma #A3853 [39]), GAPDH (Abcam #AB9484 [40]), and rabbit anti-Erk1/2 (Santa Cruz Biotechnology, #sc-93 [41]).

Membranes were washed 3 times with TBST and incubated with HRP-conjugated secondary antibodies or IRDye secondary antibodies (Li-Cor, goat anti mouse #680LT, goat anti rabbit #800CW). After 3 final washes with TBST, blots were incubated with ECL reagents (GE Healthcare), and chemiluminescent signals were detected by exposure to autoradiography film, or blots were imaged using a Li-Cor Odyssey CLX imaging system. Films were scanned using a desktop scanner, and band intensities were determined using ImageJ, or Odyssey images were analyzed using Image Studio Lite Ver 4.0. For all western blot experiments, intensity data were normalized to ERK1 as loading control. In some experiments we also probed for EGFP (rabbit anti-GFP (Invitrogen, #A11122) as a control for consistency of transduction efficiency. As shown in S1 Fig, transduction efficiency was similar in replicate PC12 cell cultures and there was relatively strong concurrence between ERK loading and GFP transfection controls. Comparable results were achieved with transfected HEK293 cells.

### Immunofluorescence

Cells were fixed for 12–15 min in 4% paraformaldehyde, washed 3 times with 1x PBS, blocked with Superblock (Thermo Scientific) supplemented with 0.3% Triton-X for 1 h at room temperature and incubated overnight at 4°C with primary antibodies. The following primary antibodies were used for immunofluorescence: mouse anti-tyrosine hydroxylase (Millipore, #MAB318), rabbit anti-tyrosine hydroxylase (Millipore, #AB152), rabbit anti-GFP (Invitrogen, #A11122), chicken anti-GFP (Invitrogen, #A10262), mouse anti-HA (Cell Signaling Technology, #2367), and rabbit anti-phospho-Akt (Thr308) (Cell Signaling Technology, #13038). Cells were washed 3 times with PBS and incubated with fluorescent secondary antibodies for 1–2 h at room temperature: AlexaFluor-568 anti-mouse, or anti-rabbit, AlexaFluor-488 anti-chicken, anti-mouse, or anti-rabbit, and AlexaFluor-350 anti-mouse or anti-rabbit (Invitrogen). For HEK293 cells grown in multiwell dishes, Hoechst 33328 was added to the first PBS wash, cells were washed 2 additional times in PBS, and cultures were observed with an inverted fluorescence microscope. For ventral midbrain dopaminergic neurons grown on glass coverslips, after 3 final washes with PBS, coverslips were mounted on slides with Vectashield mounting medium containing DAPI for nuclear staining (Vector Laboratories). Images were acquired using an Olympus inverted or upright fluorescent microscope equipped with a digital camera and Cellsens software.

### Animal use

This study was carried out in strict accordance with the recommendations in the Guide for the Use of Laboratory Animals of the National Institutes of Health. All animal procedures, described herein were approved by the Columbia University Animal Care and Use Committee, IACUC (protocols AC-AAAR7401 and AC-AAAR8404). All viral injections were carried out using ketamine/xylazine anesthesia and all efforts were made to ameliorate animal suffering. Euthanasia was carried out by CO2 inhalation followed by cervical dislocation (mice) or thoracotomy (rats) consisent with the recommendations of the Panel on Euthanasia of the American Veterinary Medical Association.

### Preparation and injection of viral vectors

#### Lentivirus

All viral plasmids were obtained from Addgene. The transfer plasmid for overexpression was pWPI, which encodes transgene-IRES-GFP under the control of the EF1-alpha promoter. Viruses were prepared in HEK293T cells using second-generation packaging plasmids pMD2G and psPAX2 (obtained from Addgene) through calcium phosphate transfection. Lentiviral supernatants were collected twice (48 h and 72 h after transfection) and concentrated using Lenti-X concentrator (Clontech, #631231) according to the manufacturer's protocols, resuspended in PBS, and stored at -80°C.

For transduction of neuronal cultures, 5–10 MOI were added directly in the medium of neuronal PC12 cells, primary cortical neurons, or ventral midbrain dopaminergic neurons. Transduced neurons were analyzed by western blot or immunofluorescence after 5–7 days.

#### Adeno-associated virus

All vectors used for these studies were AAV1 serotype. For AAV vector generation, the DD-HA-Akt(E40K) gene was modified to incorporate a Flag-encoding sequence (5’-GACTACAAAGACGATGACGACAAA-3’) at the 3’ end of the gene (denoted as DD-Akt(E40K)-Flag) and inserted into an AAV packaging construct that utilizes the chicken β-actin promoter and contains a 3′ WPRE (pBL) as previously described [20]. Similarly, an Akt(E40K) insert with no DD domain was modified to incorporate a Flag-encoding sequence and inserted into an AAV packaging construct (pBL). All nucleotide sequences in the AAV packaging constructs were confirmed before AAV production. AAVs were produced by the University of North Carolina Vector Core. The viral titer of AAV-DD-Akt(E40K)-Flag was 2.3 x 10^11^ viral molecules/ml and those of AAV-Akt(E40K) was 4.9 x 10^12^ viral molecules/ml.

For AAV injections, adult (8 week) male C57BL/6 mice were obtained from Charles River Laboratories, Wilmington, MA. Mice were anesthetized with ketamine/xylazine solution and placed in a stereotaxic frame (Kopf Instruments, Tujunga, CA) with a mouse adapter. For intranigral injections, the tip of a 5.0 μl syringe needle (26S) was inserted to stereotaxic coordinates AP: −0.35 cm; ML: +0.11 cm; DV: −0.37 cm, relative to bregma. For intrastriatal injections, the tip of a 5.0 μl syringe needle (26S) was inserted to stereotaxic coordinates AP: +0.09 cm; ML: +0.22 cm; DV: -0.25 cm relative to bregma. Viral vector suspension in a volume of 2.0 μl was injected at 0.1 μl/minute over 20 minutes.

### Oral Trimethoprim (TMP) administration

Mice were given freshly prepared 1.31 mg/ml TMP-lactate (ChemImpex International), which is equivalent of 1 mg/ml TMP, in their drinking water. TMP solutions were covered with aluminum foil to protect from light exposure and changed every 2–4 days for 3 weeks.

### Immunohistochemical staining of brains

For Flag immunostaining, mice were perfused with 0.9% NaCl for 5 min followed by 4% paraformaldehyde in 0.1 M phosphate buffer for 10 min. Perfused mouse brains were postfixed for 24 h, cryoprotected in 20% sucrose overnight, and then rapidly frozen by immersion in isopentane on dry ice. A complete set of serial sections was then cut through the SN or striatum at 30 μm. For SN sections, beginning with a random section between 1 and 4, every fourth section was chosen for immunostaining. For striatal sections, beginning with a random section between 1 and 6, every sixth section was chosen for immunostaining. Chosen sections were treated with Mouse-on-Mouse Blocking Reagent (Vector Labs) and processed free-floating with a mouse monoclonal anti-Flag antibody (Sigma) at 1:1000. Sections were incubated with biotinylated anti-mouse IgG (Vector Labs), followed by ABC (Vector Labs). After immunoperoxidase staining, sections were mounted, dehydrated, and analyzed for neuronal counts using stereological analysis.

### Stereological analysis

To determine the number of Flag-positive neurons in the SN or the striatum, the entire region of interest was defined within the StereoInvestigator program (MicroBrightField, Williston, VT). A fractionator probe was established for each section. The number of Flag-positive neurons in each counting frame was determined by focusing down through the section, using 100x objective under oil, as required by the optical dissector method. Our criterion for counting an individual Flag-positive neuron was the presence of its nucleus either within the counting frame, or touching the right or top frame lines (green), but not touching the left or bottom lines (red). The total number of Flag-positive neurons for each brain was then determined by the StereoInvestigator program.

### Statistical analysis

All statistical analyses were performed with GraphPad Prism 6 software. Simple comparisons of two experimental groups were performed using t-tests. Multiple comparisons of more than two experimental groups were performed using two-way ANOVA and Tukey’s multiple comparisons test. The threshold of significance was set at α = 0.05 for all experiments.

## Results

### Expression and inducibility of DD-Akt(E40K)

**Fig 1:**
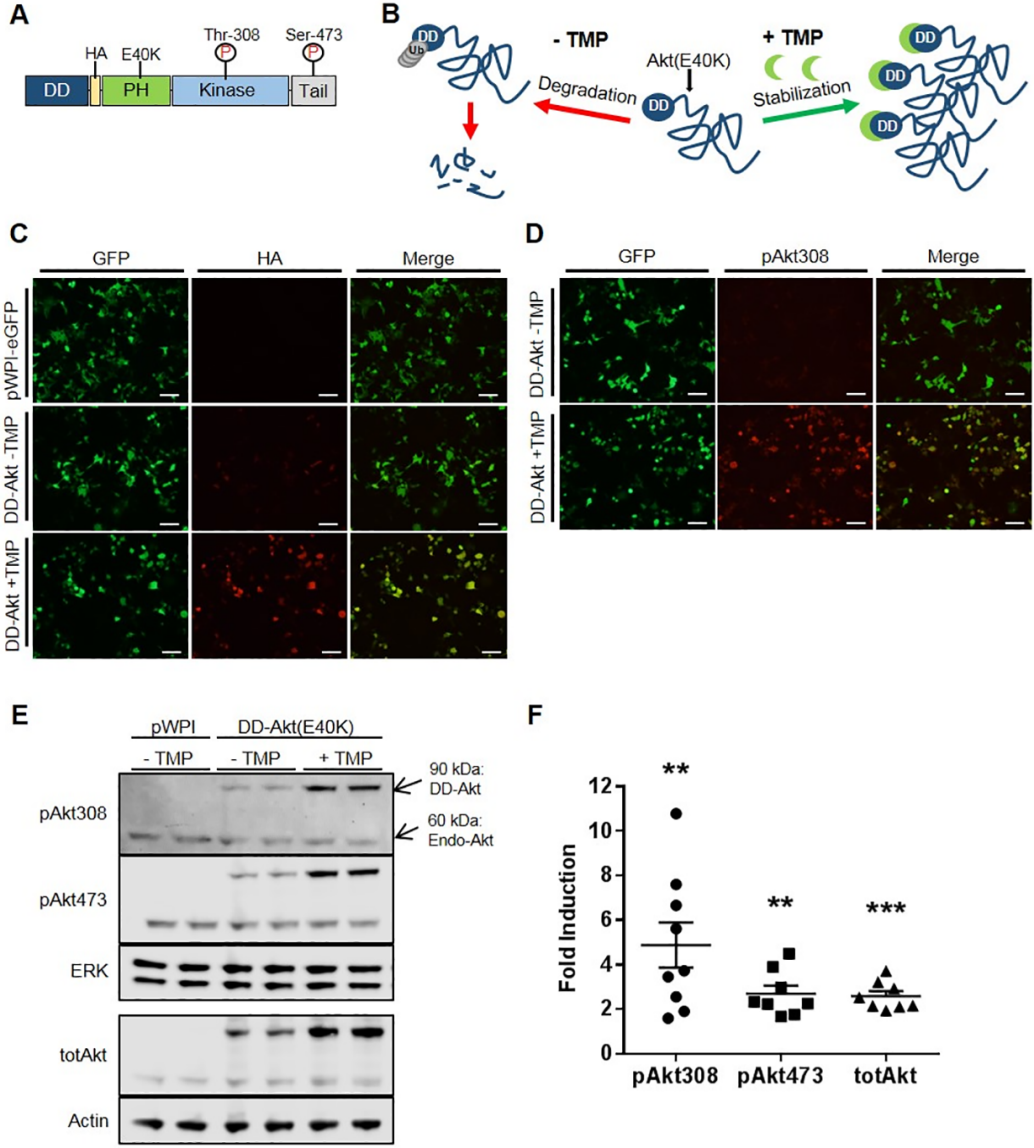
Generation and characterization of an inducible DD-Akt(E40K). (A) Design and schematic structure of the DD-Akt(E40K) fusion protein. (B) Scheme depicting mechanism of predicted induction of DD-Akt(E40K) in presence of TMP. (C) Induction of DD-Akt(E40K) by TMP in HEK293 cells as visualized by immunostaining. Cultures were transfected with a bicistronic IRES expression vector (pWPI) expressing the DD-Akt(E40K) fusion protein and eGFP, treated with or without 10 μM TMP for 24 hr and then fixed for immunofluorescence staining. GFP staining indicates cells expressing the vector while HA immunostaining reveals expression of the DD-Akt(E40K) fusion protein. Scale bars indicate 100 μm. (D) Induction of phosphorylated DD-Akt(E40K) by TMP in HEK293 cells as visualized by immunostaining. Cultures and treatment were as in (C) except that immunostaining was for pAkt(pT308) to visualize active Akt. (E) Western immunoblot analysis of total and phosphorylated DD-Akt(E40K) induction by TMP in HEK293 cells. Treatment was as in (C) with the addition that some samples were derived as indicated from cultures transfected with pWPI vector expressing only eGFP. Arrows show positions of endogenous (endo-) and DD-Akt forms. Blots were probed for total Akt and for pAkt(pT308) and pAkt(pS473). ERK 1 and actin served as loading controls. (F) Average fold induction of indicated DD-Akt forms by TMP treatment. HEK293 cultures were prepared as above and quantification of expression determined by western immunoblotting and quantification of relative band densities by ImageJ as in Methods. N = 8–9 experiments each, with duplicate or triplicate wells per condition. Graph shows means with SEM. **p < 0.01, ***p < 0.001, paired t-test +TMP vs.–TMP levels.

To create an inducibly active Akt system, we genetically fused DHFR-derived DD to the N-terminus of HA-tagged constitutively active Akt(E40K) (Fig 1A). The Akt(E40K) construct used here consists of the mouse Akt1 isoform with a glutamate to lysine substitution at amino acid 40, located within the PH domain. This substitution is analogous to the reported star mutation in the PH domain-containing Bruton tyrosine kinase and reported to enhance the binding specificity of Akt to phospholipids at the plasma membrane, thus leading to higher basal activity that is sufficient to replicate multiple biological effects associated with Akt activation [42–44]. In principle, this strategy would permit the accumulation of active DD-Akt(E40K) after treatment with the stabilizing ligand, TMP (Fig 1B).

As an initial means to validate the inducibility of the DD-Akt(E40K) fusion protein by TMP, an expression vector for bicistronic expression of both HA-tagged DD-Akt(E40K) and GFP was created and transfected into HEK293 cells. After 24 hr of TMP (10 μM) treatment, we observed markedly increased levels of HA immunostaining within cells in a distribution similar to that of GFP, indicating stabilization and accumulation of DD-Akt(E40K) (Fig 1C). There was also a low, but detectable level of HA staining in cultures transfected with DD-Akt(E40K) but untreated with TMP compared to cultures transfected with an empty vector (pWPI), indicating that there is a basal level of DD-Akt(E40K) expression without TMP.

As discussed above, Akt(E40K) has altered phospholipid binding activity leading to increased plasma membrane recruitment, which promotes its phosphorylation at Ser473 and Thr308 and consequent activation. To determine whether TMP treatment would also lead to accumulation of activated Akt, HEK293 cells were transfected as above, treated with 10 μM TMP for 24 hr, and processed for immunofluorescence staining of GFP and pAkt-T308 (Fig 1D). We observed robust induction of phosphorylated Akt staining within transfected cells after TMP treatment, thus paralleling the increased expression of stabilized DD-Akt(E40K) (Fig 1C).

The induction of total and activated Akt was also apparent when cells were processed for western immunoblotting (Fig 1E; see S2A–S2C Fig for uncropped blots with mol wt markers). With the presence of the DHFR-derived DD domain, DD-Akt(E40K) is easily distinguished from endogenous Akt in western blots due its higher molecular weight. The average fold-induction levels from multiple independent experiments were about 2.5-fold for total Akt and for pAkt-(pS473), and nearly 5-fold for pAkt(pT308) (Fig 1F). It should be noted that fold-induction was variable across experiments primarily due to differences in the detectable background levels of DD-Akt(E40K) in TMP-untreated cultures.

Because the E40K mutation of Akt increases its membrane affinity, it was conceivable that this would interfere with its degradation and, therefore, inducibility of DD-Akt(E40K). To assess this, we prepared a DD-Akt(WT) construct and compared its expression and inducibility with that of DD-Akt(E40K) by western immunoblotting of total and phosphorylated Akt forms (S3 Fig). There were no significant differences in the expression of these forms in the absence of TMP nor induction of total Akt in presence of the drug. As anticipated by its enhanced membrane association, induction of the activated phospho-Akt proteins was significantly higher for DD-Akt(E40K) compared with DD-Akt(WT).

We also evaluated the possibility that the presence of an additional DD domain will reduce basal expression of the Akt(E40K) fusion protein in absence of TMP by promoting more efficient degradation. In designing such a construct, we further considered that the close proximity of the DD-domain to the much larger Akt protein might hinder degradation. Our initial DD-Akt(E40K) construct contains an Arg-Ala-Cys linker between the DD domain and the HA-tag. We therefore assessed various constructs (S4 Fig) with a second DD-domain and glycine linker sequences (Gly-Gly-Gly-Gly-Ser)_1-2_ (G_4_S)_1-2_ with the potential for enhanced flexibility as previously described (Iwamoto et al., 2010). We found that such constructs did not undergo substantially different levels of expression or induction by TMP compared with DD-Akt(E40K), with the only apparent significant difference being an approximate 60% increase in induction of (DD-G_4_S)_2_-pAkt(E40K-pT308) by TMP (S4 Fig).

Given that the E40K mutation did not interfere with induction of the total protein by TMP and enhanced induction of the active forms, and that the modified constructs did not confer substantially enhanced levels of TMP induction, subsequent experiments were carried out with the initial DD-Akt(E40K) construct.

### DD-Akt(E40K) is inducible in a neuronal background

**Fig 2:**
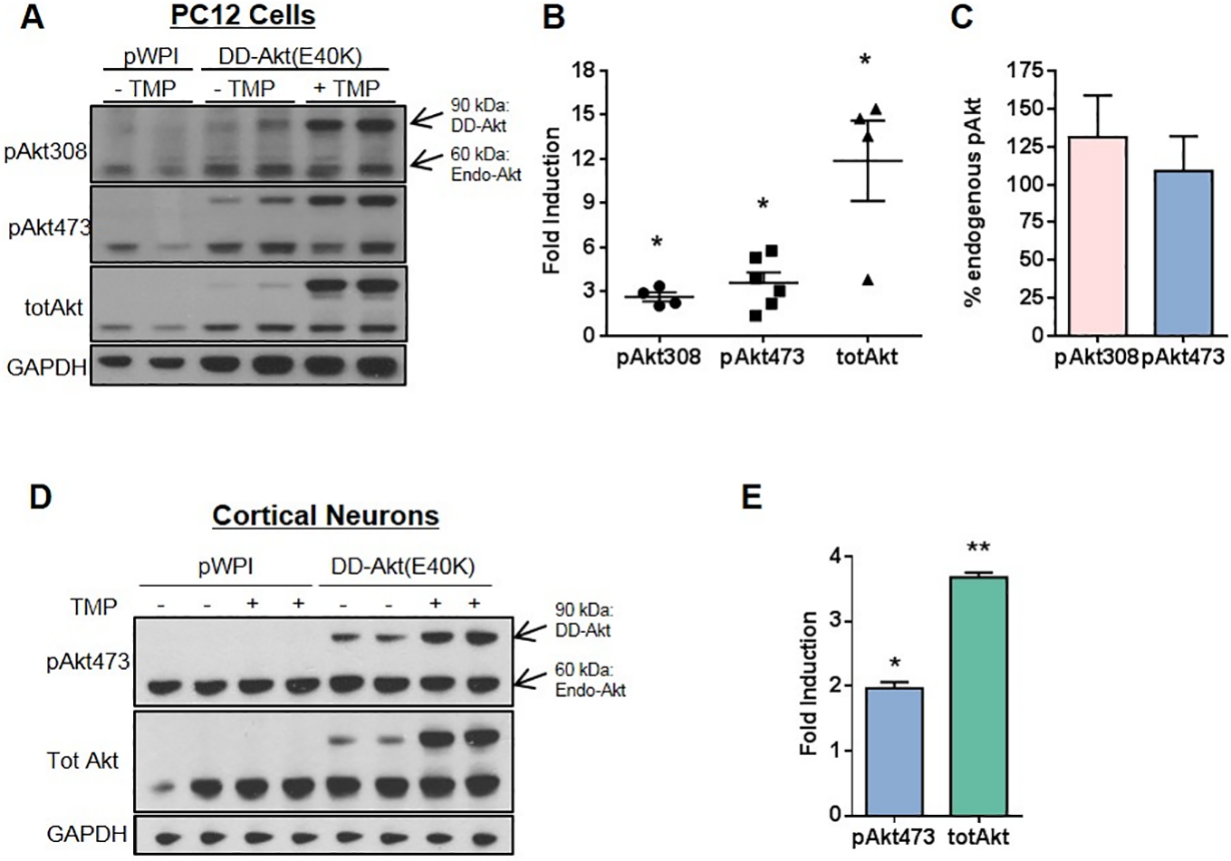
DD-Akt(E40K) is inducible in a neuronal context. (A) Western immunoblot analysis of DD-Akt(E40K) induction by TMP in neuronal PC12 cells. Neuronal PC12 cells were infected as indicated with a lentiviral vector expressing either eGFP alone (pWPI) or the DD-Akt(E40K) fusion gene and eGFP. Four days post-infection, cells were treated with or without 10 μM TMP as indicated for 24 hr and then harvested for western immunoblotting. Blots were probed as indicated for total Akt, pAkt(pT308), pAkt(pS473) and GAPDH (loading control). A representative blot is shown. (B) Average fold induction of DD-Akt forms by TMP treatment in neuronal PC12 cultures. N = 4–6 independent experiments with duplicate or triplicate wells per condition. Graph shows means with SEM. *p ≤ 0.05, paired t-test, +TMP vs.–TMP levels. (C) The relative expressions of induced DD-pAkt(E40K) and endogenous pAkt levels in neuronal PC12 cells quantified and calculated as a percent ratio. (D) Western immunoblot analysis of total and phospho-S473 forms of DD-Akt(E40K) induction by TMP in cultured rat cortical neurons. Cultured rat cortical neurons were infected as indicated with a lentiviral vector expressing either eGFP alone (pWPI) or the DD-Akt(E40K) fusion gene and eGFP. Four days post-infection, cells were treated with or without 10 μM TMP as indicated for 24 hr and then harvested for western immunoblotting. Blots were probed as indicated for total Akt, pAktT308, pAktS473 and GAPDH (loading control). Assessment of GFP expression indicated an infection efficiency of about 50% of the neurons. A representative blot is shown. (E). Average fold induction of total and DD-pAkt(E40K-pS473) by TMP treatment in cortical neuron cultures. N = 2 samples per condition. Values are means with SEM. *p<0.05, **p<0.01, unpaired t-test, +TMP vs -TMP.

The inducibility of DD-Akt(E40K) was further tested in a different cell type, neuronally-differentiated PC12 cells. These have been widely used to model neurons and have been employed in numerous studies of potential relevance to neurotrophic factor signaling and to PD and other neurodegenerative disorders [18, 29, 32, 45]. The cells were transduced with either empty or DD-Akt(E40K)-expressing lentiviral vectors, treated with or without 10 μM TMP for 24 hr, and then processed for immunoblotting to determine levels of DD-Akt(E40K) expression (Fig 2A and 2B). Here again, induction by TMP was evident with mean levels of increase of 2.6-fold for DD-pAkt(E40K-pT308), 3.6-fold for DD-pAkt(E40K-pS473) and 11.9-fold for total DD-Akt(E40K). We also compared the levels of DD-pAkt(E40K) reached after TMP induction to those of endogenous pAkt (Fig 2C). This revealed that both DD-pAkt(E40K-pT308) and DD-pAkt(E40K-pS473) reached levels comparable to those of the corresponding endogenous proteins. Although transduction efficiency was not assessed in all experiments, efficiency was approximately 75% for our experimental conditions as measured by the proportion of GFP-expressing cells.

The inducibility of DD-Akt(E40K) also was assessed in primary cultures of rat cortical neurons. Four days after infection with lentivirus expressing GFP alone or DD-Akt(E40K) and GFP, the cultures were treated for 24 hr with or without 10 μM TMP and evaluated by western immunoblotting for levels of total and phosphorylated (at S473) Akt. There was an approximate 2-fold induction by TMP of DD-pAkt(E40K-pS473) and a 3.5-fold increase of total DD-Akt(E40K) (Fig 2D and 2E). Assessment of GFP expression indicated an infection efficiency of about 50% of the neurons. Taking this into consideration, the data indicate that the levels both of total and phosphorylated DD-Akt reached after induction were comparable or greater than the endogenous levels of the corresponding proteins (Fig 2D).

### DD-Akt(E40K) inducibility is dose-responsive and reversible

**Fig 3:**
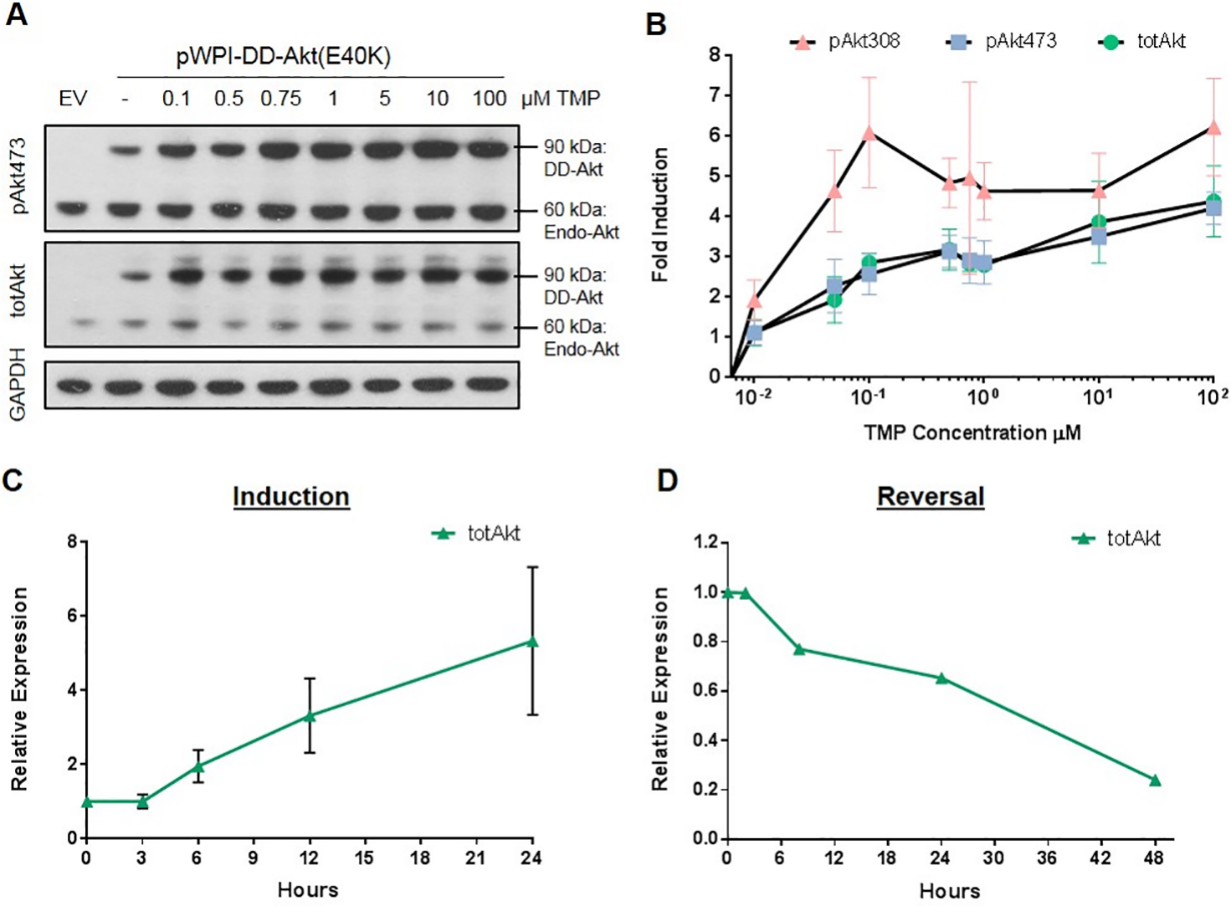
DD-Akt(E40K) induction is dose-responsive to TMP treatment and reversible. (A) Western immunoblotting analysis of dose-response of DD-Akt(E40K) induction by TMP. HEK293 cells were transfected with eGFP-only expressing vector (EV) or DD-Akt(E40K) fusion protein expression vector and treated with indicated doses of TMP for 24 hr before analysis by western immunoblotting with the indicated probes. (B) Quantification of DD-Akt(E40K) induction (as determined by western immunoblotting) by various doses of TMP. Values means with SEM. N = 3–4 independent experiments. (C) Time course of DD-Akt(E40K) induction by TMP. HEK293 cells were transfected with DD-Akt(E40K) expression vector, treated with 10 μM TMP for the indicated times and then assessed for total DD-Akt(E40K) levels by western immunoblotting as in (A). Values are means with SEM. N = 2–4 independent experiments. (D) Reversal of DD-Akt(E40K) induction after TMP washout. HEK293 cultures transfected with DD-Akt(E40K) as above were treated with 10 μM TMP for 24 hr, washed free of TMP and harvested for western immunoblotting at the indicated time points. Total DD-Akt(E40K) levels were determined by western immunoblotting as in (A). Data shown are from one experiment.

One of the potential advantages of the DD system is its dose-responsive inducibility by the stabilizing ligand [22]. To assess this for our construct, HEK293 cells were transiently transfected with DD-Akt(E40K) expression vector and then treated with doses of TMP ranging from 10 nM to 100 μM for 24 hr. The induction of DD-Akt(E40K) and DD-pAkt proteins was dose-responsive to TMP, reaching half-maximal induction by approximately 30 nM (Fig 3A and 3B). We also examined the kinetics of induction and reversal of DD-Akt induction by TMP. DD-Akt(E40K)-transfected HEK293 cells were assessed by western blotting at different times after either TMP addition or washout. Induction of DD-Akt(E40K) by TMP was evident by 6 hr and appeared to continue to accumulate up to at least 24 hr. (Fig 3C). To assess reversal, transfected cells were treated with 10 μM TMP for 24 hr, washed free of the drug and analyzed by western immunoblotting at various time points (Fig 3D). This showed a nearly linear decrease of DD-Akt(E40K) levels with approximately 30 hr required for half reversal of induction.

### DD-Akt(E40K) induction leads to elevated phosphorylation of downstream substrate FoxO4

**Fig 4:**
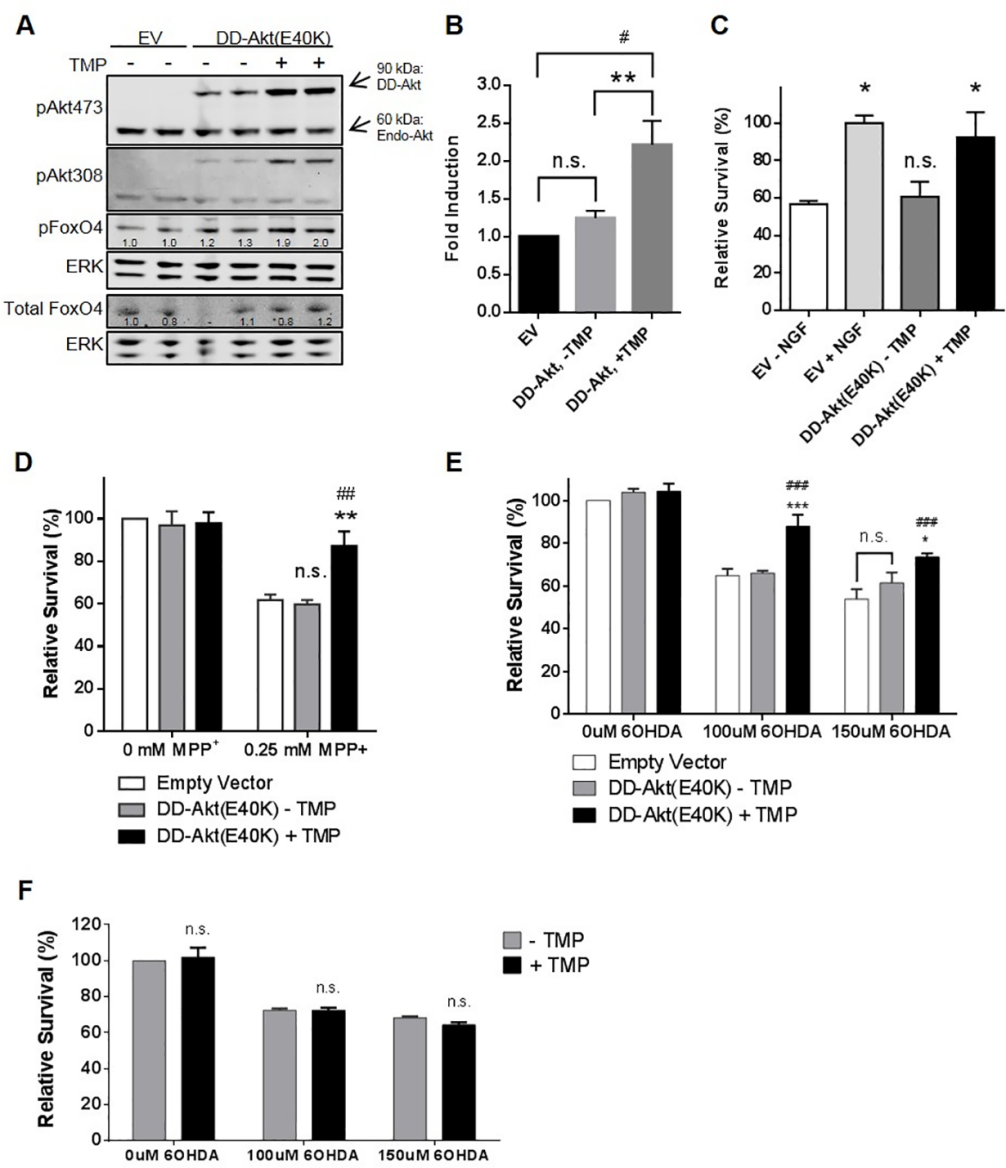
Induction of DD-Akt(E40K) leads to substrate phosphorylation and neuroprotection. (A) Induction of DD-Akt(E40K) promotes phosphorylation of the putative Akt substrate FoxO4 on pS193. HEK293 cells were transfected with vector expressing eGFP alone (EV) or DD-Akt(E40K) and eGFP, treated with or without 10 μM TMP for 24 hr, and then harvested and analyzed by western immunoblot analysis for the indicated proteins. Total FoxO4 was analyzed on a separate immunoblot and is shown with its own loading controls. Numbers below the bands indicate relative densities (determined by ImageJ), normalized to corresponding ERK1 loading control. (B) Quantification of western immunoblotting data for effect of DD-Akt(E40K) induction on pFoxO4(pS193) levels. Experiments were performed and quantified as in (A). Values are given as mean fold-induction of pFoxO4(pS193) levels under the indicated conditions with SEM. N = 9 independent experiments (7 experiments with no serum present, 2 experiments with serum present). **p<0.01 compared with DD-Akt(E40K)—TMP condition, #p<0.05 compared with empty vector (EV) condition, one-way ANOVA. (C) DD-Akt(E40K) induction protects against serum deprivation. Naïve (NGF-untreated) PC12 cells were infected with lentiviral vectors for overexpression of DD-Akt(E40K) and eGFP or eGFP alone (EV). Four-five days after infection, cultures expressing DD-Akt(E40K) were pre-treated with or without 10 μM TMP for 24 hr and then extensively washed and replated with serum-free medium with or without the continued presence of TMP. EV cultures were deprived of serum in similar fashion and treated either with or without NGF (100 ng/ml). After 16–24 hr of serum deprivation, cells were lysed for quantification of viable nuclei to measure survival. Average infection efficiency was 60%. Data were normalized to survival in NGF-treated cultures and are expressed as means with SEM. *p < 0.05, n.s. vs. EV-NGF; 2-way ANOVA. N = 2–3 independent experiments. (D) Induction of DD-Akt(E40K) protects against the PD mimetic toxin MPP+. Neuronal PC12 cells were infected with lentiviruses for overexpression of DD-Akt(E40K) and eGFP or eGVP alone (EV). Four to five days after infection cultures were pre-treated with or without 10 μM TMP for 24 hr before addition of 0.25 mM MPP+. After 18–24 hr of MPP+ treatment, cells were lysed for quantification of viable nuclei to measure survival. Values are means with SEM and were normalized to cell numbers in cultures infected with EV and not exposed to MPP+. **p < 0.01 vs.–TMP; ##p < 0.01 vs. EV; n.s. vs. EV; 2-way ANOVA. N = 3 independent experiments. (E) Induction of DD-Akt(E40K) protects against the PD mimetic toxin 6-OHDA. Neuronal PC12 cell cultures were established and treated and analyzed as above, except that treatment was with 6-OHDA. Values are means with SEM. ***p < 0.001 vs.–TMP; *p < 0.05 vs.–TMP; ### p < 0.001 vs. Empty Vector; n.s. vs. Empty Vector; 2-way ANOVA. N = 3 independent experiments. (F) TMP does not protect from 6-OHDA in absence of DD-Akt(E40K). Neuronal PC12 cells were pretreated with or without 10 μM TMP for 24 hr before addition of 100 μM or 150 μM 6-OHDA and assessed as above for survival 18–24 hr later. Values are means with SEM. There were no significant effects of TMP on cell survival under any conditions, 2-way ANOVA. n = 2 independent experiments with 3 replicates per condition per experiment.

To validate that the induced DD-Akt(E40K) fusion protein maintains its function as a kinase, we investigated the effect of its induction on a purported Akt substrate, the transcription factor FoxO4 [1]. HEK293T cells were transfected with DD-Akt(E40K) expression vector, treated with or without 10 μM TMP for 24 hr, and analyzed by western immunoblotting for levels of FoxO4 phosphorylated at the phosphorylation site linked to Akt-dependent phosphorylation, S193 (pFoxO4) (Fig 4A and 4B). This revealed an approximate doubling of pFoxO4 levels after DD-Akt(E40K) induction with TMP. In contrast, there was no significant elevation of pFoxO4 after DD-Akt(E40K) transfection in the absence of TMP treatment, thus indicating that basal expression levels of DD-Akt(E40K) were insufficient to significantly elevate FoxO4 phosphorylation (pS193). Additionally, under the various experimental conditions, there was no change of total FoxO4 levels (Fig 4A).

### TMP-mediated induction of DD-Akt(E40K) protects against cell death

We next examined whether DD-Akt(E40K) induction promotes physiologic responses. Undifferentiated (NGF-untreated) PC12 cells rely on the presence of serum in the medium for survival. When serum is removed from the medium, the cells undergo apoptosis that can be fully reversed by the addition of NGF [46]. This action of NGF appears to be dependent on Akt signaling [47]. We therefore used this model to provide a functional readout for TMP-induced DD-Akt(E40K) induction.

PC12 cells were transduced with lentiviral vectors to co-express DD-Akt(E40K) and GFP (or empty vector and GFP) and subsequently treated with 10 μM TMP for 24 hr. Cells were washed free of serum and replated in serum-free medium under continued presence or absence of TMP. Cultures infected with empty vector and treated with NGF were included as positive controls for normalization. Counts of surviving cells were performed after 16–24 hr of serum deprivation (Fig 4C). Under these serum-free conditions and when compared with NGF-treated cultures, a mean of 57% ± 2% of cells survived in cultures transduced with empty vector and not treated with NGF. In contrast, survival in cultures transduced with DD-Akt(E40K) and treated with TMP averaged 92% ± 13% of survival reached with NGF. Cultures transduced with DD-Akt(E40K) but not induced with TMP treatment had a mean survival rate of about 61% ± 8%, which was not significantly different from that in control NGF-untreated cultures. Taken together, these findings indicate that induction of DD-Akt(E40K) by TMP treatment is sufficient to protect PC12 cells from serum deprivation-induced apoptosis to a degree similar to that achieved with NGF, and that the level of DD-Akt(E40K) expressed without induction is insufficient to provide significant protection.

### Induced DD-Akt(E40K) protects against cell death evoked by MPP+ and 6-OHDA

6-hydroxydopamine (6-OHDA) has been widely used in *in vitro* and *in vivo* models of PD as have 1-methyl-4-phenyl-1,2,3,6-tetrahydropyridine (MPTP) and its metabolite 1-methyl-4-phenylpyridinium (MPP^+^) [48]. Both neurotoxins cause selective degeneration of dopaminergic neurons, and MPP^+^ and 6-OHDA can induce degeneration of neuronal PC12 cells, providing an *in vitro* model that is potentially relevant to PD [49, 50]. Prior work indicates that activation of Akt protects neurons from these toxins both *in vitro* and *in vivo* [18, 51]. To determine whether DD-Akt(E40K) can inducibly protect neuronal PC12 cells from these PD mimetic toxin insults, we infected cultures with DD-Akt(E40K) expressing lentivirus or with an empty virus and then pretreated with or without 10 μM TMP for 24 hr before exposure to 0.25 mM MPP^+^, or 100–150 μM 6-OHDA for 18–24 hr. The numbers of surviving cells were then determined under each condition. As shown in Fig 4D and 4E, there was significant protection in both models in TMP-treated cultures expressing DD-Akt(E40K). In contrast, there was no significant protection for corresponding cultures not treated with TMP, indicating that the basal expression of DD-Akt(E40K) was insufficient to impact survival rates. The transduction efficiency for these cultures was approximately 75%, as determined by immunofluorescence staining of GFP-positive cells. If a correction is made for incomplete transduction, the survival with TMP-induced DD-Akt(E40K) would be 95% in the MPP^+^ toxin model, 96% at the lower dose of 6-OHDA, and 81% at the higher dose of 6-OHDA.

To verify that the observed protection after TMP treatment was not due to the drug alone, we pre-treated uninfected neuronal PC12 cell cultures with or without 10 μM TMP for 24 hr and then assessed survival after 18–24 hr of exposure to 100 or 150 μM 6-OHDA. As shown in Fig 4F, TMP alone showed no significant effect on cell death in this model.

### Inducibility of DD-Akt(E40K) appears to be region-specific *in vivo*

**Fig 5:**
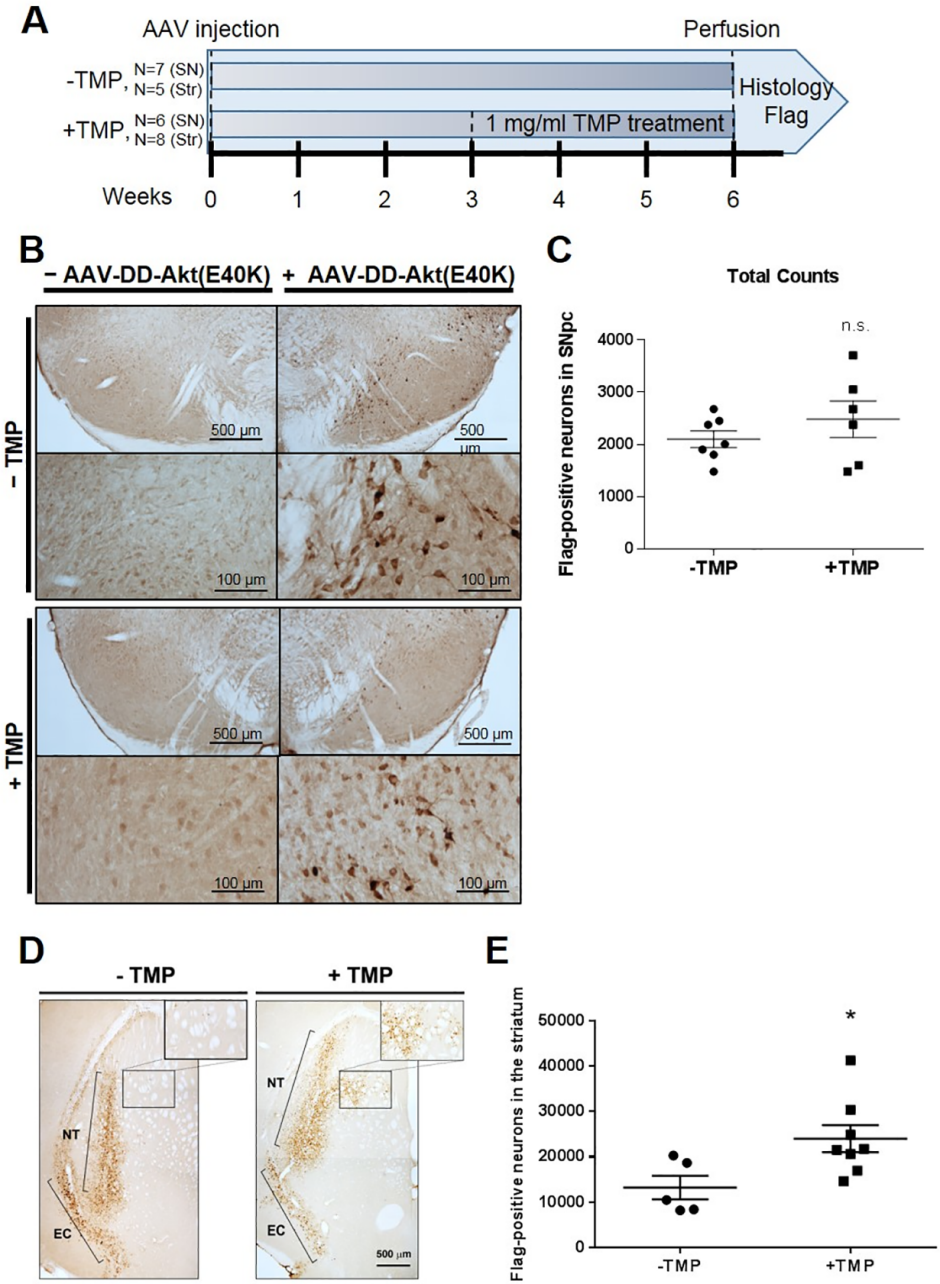
Regional inducibility of DD-Akt(E40K) in the mouse brain. (A) Scheme of experimental design. Adult C57BL/6 mice were subjected to unilateral AAV-DD-Akt(E40K)-Flag injections into the SNpc or striatum. At 3 weeks post-viral injection, the mice were treated with or without 1 mg/ml TMP in their drinking water for 3 weeks and then their brains were harvested and processed for Flag immunostaining. (B) Representative images of Flag immunostained neurons in the SNpc of both TMP-untreated and TMP-treated mice. (C) Stereological quantification of total numbers of Flag-stained neurons in the SNpc of mice treated as above with or without TMP. The data represent total Flag-positive neuron numbers in the SNpc per mouse. Mean values are shown with SEM. n = 7 mice for -TMP, n = 6 mice for +TMP. n.s., determined by unpaired t-test. (D) Representative images of Flag immunostaining in the striatum of both TMP-untreated and TMP-treated mice. Staining in the medial striatum is outlined by a black rectangle and shown at higher magnification in the inset; the external capsule (EC) and needle track (NT) staining is indicated by brackets. (E) Stereological quantification of total numbers of Flag-stained neurons in the striata of mice treated as above with or without TMP. The data represent total Flag-positive neuron numbers in the entire striatum per mouse. Mean values are shown with SEM. n = 5 mice for -TMP, n = 8 mice for +TMP. *p = 0.03, unpaired t-test (+TMP vs -TMP).

The observations that induction of DD-Akt(E40K) is protective in *in vitro* PD models and that virally-delivered myr-Akt protects dopaminergic neurons of the substantia nigra in a 6-OHDA model [20] prompted us to examine whether DD-Akt(E40K) might be suitable as an inducible neuroprotective agent for dopaminergic neurons in living animals. Towards this end, we tested the inducible expression of DD-Akt(E40K) in two regions of the mouse brain: the SNpc and striatum. A Flag-tagged DD-Akt(E40K) construct was packaged into an adeno-associated virus serotype 1 (AAV1) vector that selectively infects neurons [20] and stereotactically delivered unilaterally to the SNpc or striatum of adult male C57BL/6 mice following previously described procedures. We confirmed in HEK293 cells that the Flag-tagged construct was inducible similarly to the HA-tagged construct (data not shown). Because TMP passes the blood brain barrier [52], the drug was administered in the drinking water, beginning at 3 weeks post-injection to allow for expression of the transgene. Previous literature has described the induction of DD-fusion proteins in the rat striatum with a TMP dose range of 0.1–0.5 mg/ml, administered in the drinking water for 3 weeks [22, 26, 27]. In our study, the mice were treated for 3 weeks with or without 1 mg/ml TMP in the drinking water, and then their fixed brains were processed for Flag immunostaining as a direct readout of DD-Akt(E40K)-Flag expression (Fig 5A).

Visible observation revealed no evident difference in numbers or staining intensity of Flag-stained neurons in the SNpc of mice with or without TMP treatment (Fig 5B). Stained sections were subsequently analyzed stereologically in a blinded manner to determine total numbers of Flag-positive neurons in the SNpc of control and TMP-treated mice (Fig 5C). There was no statistically significant difference in the numbers of Flag-positive neurons between the treatment groups, indicating that expression of DD-Akt(E40K)-Flag in the mouse SNpc is not induced after oral administration of TMP.

To explore the possibility of a region-dependent effect on the induction of DD-Akt(E40K), we next examined the inducibility of the construct in the striatum of adult male mice, following the same TMP treatment protocol (Fig 5A). As noted above, past work has described control of DD-fusion proteins in rodent striatum by oral TMP administration [22, 26–28]. Although both treatment groups exhibited Flag-positive neurons, indicating that DD-Akt(E40K)-Flag was expressed, the staining in non TMP-treated mice appeared to be limited to the needle track and along the external capsule of the striatum (Fig 5D). In contrast, Flag staining in TMP-treated mice appeared to be more diffuse along the needle track in all mice and show a distinct area of staining in the medial striatum in four of seven mice. Neither of these features was observed in TMP-untreated mice. To quantify the effect of TMP treatment, sections were stereologically analyzed in a blinded manner for total numbers of Flag-positive cells within each striatum (Fig 5E). We found a statistically significant difference between treatment groups with TMP-untreated mice exhibiting a mean of 13,238 ± 2602 Flag-stained neurons per striatum and TMP-treated mice exhibiting a mean of 24,009 ± 2984 Flag-stained neurons (*p = 0.03). This translates to a 1.8-fold increase in DD-Akt(E40K) expression after TMP treatment.

### Cell type-dependent induction of DD-Akt(E40K) in ventral midbrain cultures

**Fig 6:**
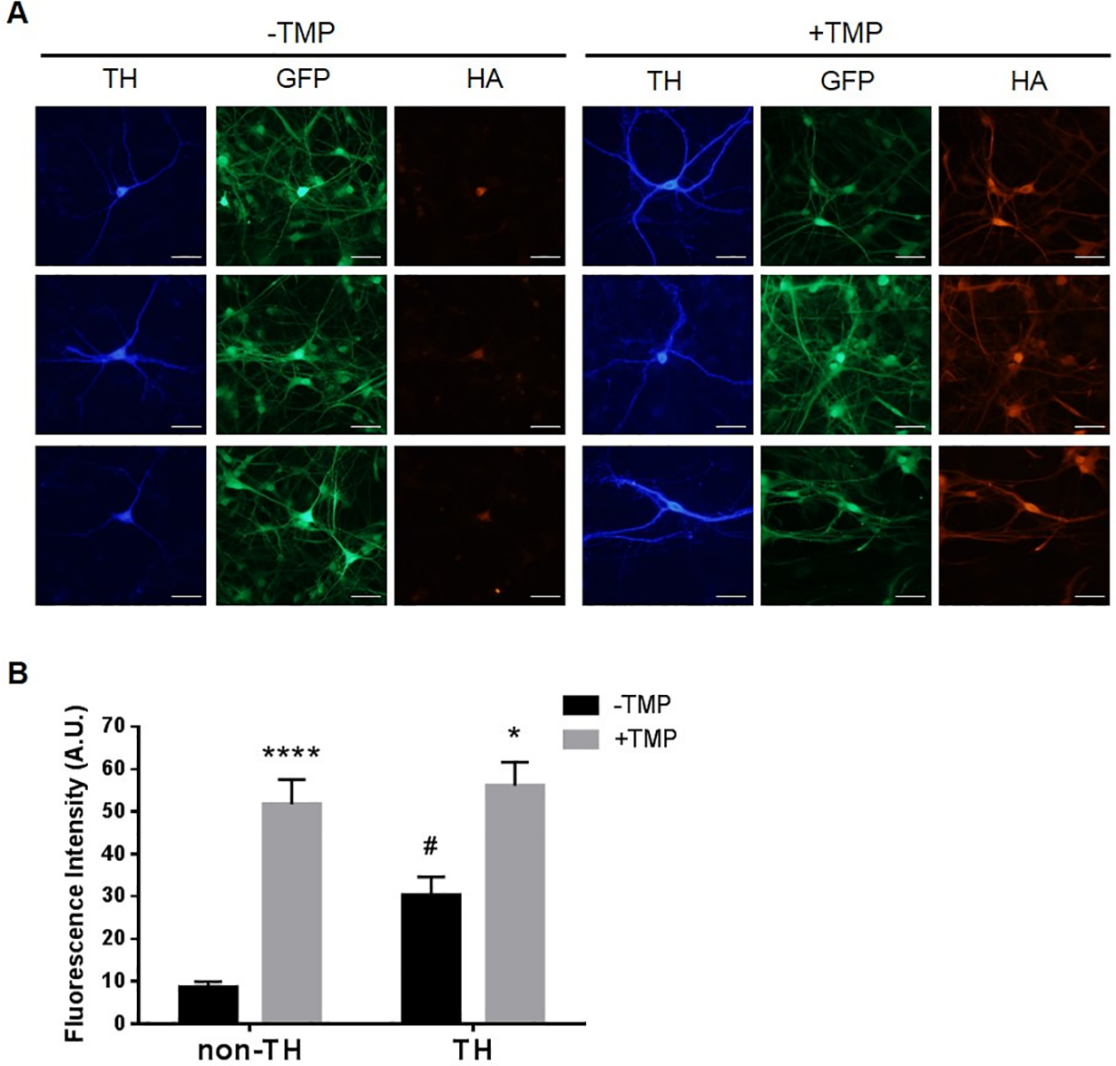
Differential induction of DD-Akt(E40K) within ventral midbrain cultures. (A) DD-Akt(E40K) induction in ventral midbrain cultures. Ventral midbrain cultures were infected with a lentiviral vector expressing DD-Akt(E40K) and eGFP. 6–10 days post-infection, cultures were treated with or without 10 μM TMP for 4 days and then processed for immunofluorescent staining of tyrosine hydroxylase (TH) to identify dopaminergic neurons, eGFP to identify infected cells, and HA to reveal DD-Akt(E40K). Scale bar indicates 50 μm. (B) HA fluorescence intensity was measured in infected (GFP+) neurons using ImageJ. Dopaminergic vs. non-dopaminergic cells were identified by TH staining. Values are mean fluoresence intensity in arbitary units (A.U.) with SEM. n = 7–8 TH+ neurons measured for each condition, 12–14 non-TH neurons measured for each condition. *p < 0.05: TH, -TMP vs. + TMP; ****p < 0.0001: non-TH, -TMP vs. + TMP; #p < 0.05: TH, -TMP vs. non-TH, -TMP, 2-way ANOVA.

The results presented above suggest that there may be region- and/or cell-type specific induction of DD-Akt(E40K). To investigate this further in a system relevant to dopaminergic neurons and PD, we examined the inducibility of DD-Akt(E40K) in primary cultures of the ventral midbrain. Ventral midbrain neurons were isolated and cultured as previously described [31] from P0-P3 rats and infected with lentivirus expressing both HA-tagged DD-Akt(E40K) and GFP. Six to 10 days post-infection, the cultures were treated with or without 10 μM TMP for 4 days and triple-stained for tyrosine hydroxylase (TH) to identify DA neurons, GFP to identify transduced cells, and HA to identify levels of DD-Akt(E40K) (Fig 6A). Neurons exhibiting dual fluorescence signals for both GFP and TH were quantified for HA signal intensity. The results show a significant increase of 1.9-fold ± 0.2 (p < 0.005 vs.-TMP) in HA fluorescence intensity for DA neurons treated with TMP compared with those without TMP exposure (Fig 6B). This indicates that under our culture conditions, DA neurons undergo TMP-dependent induction of DD-Akt(E40K).

In addition to TH+ neurons, ventral midbrain cultures contain substantial numbers of TH- neurons. These were also infected by our lentivirus and their expression of DD-Akt(E40K) appeared to be TMP-responsive (Fig 6A). Quantification of the HA signal in TH- neurons (randomly chosen in the same cultures in which the HA signals of TH+ neurons were quantified) revealed a 6-fold induction of DD-Akt(E40K) by TMP (Fig 6B). To better understand the basis for this more than 3-fold higher level of induction in TH- compared with TH+ neurons, we compared the levels of HA fluorescence intensity in the two populations. This revealed that while the mean level of signal reached after TMP treatment was similar for TH+ and TH- neurons, the level of expression without TMP was about 3-fold higher in TH+ neurons (Fig 6B). Because the expression of DD-fused proteins in absence of TMP is regulated by proteasomal degradation, these findings suggest that dopaminergic neurons in our cultures were substantially less efficient in protein turnover than their non-dopaminergic neighbors.

## Discussion

The present study describes the generation and characterization of a conditionally inducible, active form of Akt1, DD-Akt(E40K). To achieve this, we used the TMP-regulated destabilizing domain approach initially described by the laboratory of Dr. Wandless [22]. This technique has been used for a variety of applications both *in vitro* and *in vivo* [23–28, 53, 54]. Our findings indicate that DD-Akt(E40K) is: robustly induced by the ligand TMP; enzymatically active with inducibly enhanced phosphorylation of the known Akt substrate FoxO4; biologically active with promotion of serum-free cell survival and protection from the PD-mimetic toxins 6-OHDA and MPP^+^. Moreover, induction was observed in a range of cultured cells including human 293 cells, undifferentiated and differentiated rat PC12 cells, rat cortical neurons and ventral midbrain dopaminergic and non-dopaminergic neurons. We further found induction *in vivo* in mouse striatal cells. Using cultured cells, we observed that induction was dose-responsive and reversible. Moreover, expression of DD-Akt(E40K) and its phosphorylated forms was easily detectable and quantifiable by western immunoblotting. Addition of HA or FLAG tags permitted visual detection of the protein by immunostaining.

One of the potentially attractive features of the destabilizing domain approach is that the DD-fused protein is rapidly degraded in absence of the TMP ligand, thus providing a very low basal level of expression. This has provided impressive levels of induction [22]. In the present study, we observed readily detectable levels of total and phosphorylated DD-Akt(E40K) expression in absence of TMP for each of the cultured cell types that we studied. While there was consistent induction with TMP, the fold-induction observed reflected the -TMP background, providing an induction range of about 2-10-fold for total DD-Akt(E40K) and about 2-5-fold for p-DDAkt(E40K), depending on cell type. As described above, adding a second DD domain or manipulating linker configuration did not appreciably reduce expression in absence of TMP.

Because the level of endogenous Akt is not altered in DD-Akt(E40K)-transduced cells, we were also able to compare the non-induced and induced levels of the fusion protein to expression of endogenous Akt. This revealed some degree of variation with cell type. For 293 cells, the basal levels of total DD-Akt(E40K) were approximately twice those of the endogenous protein and reached 3-5-fold higher levels than the endogenous Akt after TMP treatment. In contrast, for neuronal PC12 cells and cortical neurons, total DD-Akt(E40K) was substantially less (approximately 1/3-1/2) when compared with endogenous protein without TMP, and reached levels that were 1-2-fold (cortical neurons) or about 3-fold higher (PC12 cells) to endogenous Akt after TMP treatment. In both neuronal cell types, basal phospho-DD-Akt(E40K) levels were substantially below those of endogenous p-Akt, and after induction reached levels approximately double those of endogenous p-Akt. These observations are consistent with our findings that DD-Akt(E40K) was able to protect PC12 cells from serum deprivation and PD-mimetic toxins after induction with TMP.

The ability of induced DD-Akt(E40K) to promote survival in different neuronal backgrounds suggested its potential application for prevention of neurodegeneration *in vivo*. Parkinson's disease is a particularly relevant target. Affected dopaminergic neurons in the disease show greatly diminished levels of p-Akt [18, 19] while over-expression of myristoylated Akt provides profound protection of dopaminergic cell bodies and axons in a mouse model of PD [20, 21]. Such observations suggested that a tunable and reversible Akt expression system could be of therapeutic value in preventing dopaminergic neuron degeneration in PD. However, our findings suggest that at least in the mouse substantia nigra, DD-Akt(E40K) is not induced by TMP. This contrasted with our finding of inducibility in the striatum. Several other groups have reported induction of DD-linked proteins in the striatum with TMP [27, 28], and these outcomes, in agreement with our own findings, suggest that failure of induction in the substantia nigra is not due to an experimental issue, but rather to the cellular context.

One possible reason for the lack of detectable induction of DD-Akt(E40K) in the substantia nigra might be a relatively high level of basal expression in absence of TMP. This possibility is supported by our *in vitro* studies in which dopaminergic midbrain neurons displayed a considerably higher basal level of DD-Akt(E40K) expression (and, as a result, lower induction) compared with non-dopaminergic neurons in the same cultures. If this explanation is correct, then our findings suggest that dopaminergic neurons may have lower capacity for protein degradation than other neuron types, resulting in accumulation of DD-Akt(E40K) even when TMP is not present. There is much evidence that neurodegeneration in PD is associated with inadequate degradation and consequent accumulation of misfolded and aggregated proteins [55, 56], and there are reports of selectively compromised protein degradation by dopaminergic neurons in this disorder [57, 58]. Our findings raise the possibility that dopaminergic neurons are less efficient than several other neuron types in protein clearance. If so, this in turn suggests a potential reason for the selective susceptibility of dopaminergic neurons in PD.

Our data indicate that cellular context plays an important role in determining the basal and induced levels of DD-Akt(E40K). Although dopaminergic neurons may not provide the optimal cellular environment for DD-Akt(E40K) induction, our findings show relatively low basal expression and robust induction in several other cell types. In 293 cells, induction of DD-Akt(E40K) was correlated with enhanced phosphorylation of FoxO4 and in PC12 cells correlated with promotion of survival in multiple models. Thus, in appropriate cell types, DD-Akt(E40K) may be a useful tool to interrogate the molecular, physiologic and pathologic effects of elevating Akt levels and activity in a tunable, reversible and selective manner. Induction of DD-Akt(E40K) in the striatum additionally supports the idea of using such a reagent *in vivo* and potentially, for therapeutic purposes. For instance, there is evidence linking defective Akt signaling with insulin resistance [59]. In the nervous system, Akt dysfunction has been associated with a variety of mood disorders [12, 15, 16] and Akt over-expression with promotion of CNS axon regeneration and neuron survival after injury [60]. Given the pathophysiological consequences of dysregulated Akt activity in the CNS, DD-Akt(E40K) (and related constructs) may represent an approach to enhance Akt function in disorders associated with diminished Akt expression and/or activity.

## Supporting information

S1 Fig Comparison of loading controls for transduction experiments. Upper image shows western blot of EGFP (GFP) and ERK loading controls for an experiment in which replicate cultures of PC12 cells were infected with lentivirus expressing either DD-Akt(E40K) or DD_2_-Akt(E40K) as indicated, and treated for 24 hr with or without TMP (10 μM) or 2xTMP (20 μM) and then lysed. The infection efficiencies were 78.3% and 75.6%, respectively as determined by immunofluorescence imaging for EGFP. Blots were probed for ERK and EGFP (GFP) proteins and relative intensities of the indicated bands were determined as described in Materials and Methods. Relative intensities are given in Arbitrary Units (A.U.) for each individual replicate for both GFP and ERK1 as well as the ratios thereof. Mean values for each transfection condition (DD-Akt or DD_2_-Akt) are given in italics ± S.E.M. and indicate consistency of transfection from replicate to replicate and of the GFP/ERK1 ratio.
(TIF)

S2 Fig **Uncropped blots with molecular weight markers for Fig 1E (panels A-C) and 4A (panels D,E).** Blot for total Akt is more highly exposed than in Fig 1 to show background. In each panel 6 irrelevant lanes to the right are not included.
(TIF)

S3 Fig E40K mutation does not affect DD-mediated destabilization of DD-Akts. HEK293 cells were transfected with DD constructs with WT Akt or Akt(E40K). Cells were treated with 10 μM TMP for 24 hr and then lysed for western blotting. Protein expression levels were quantified and normalized to ERK1 as a loading control. Fold induction was calculated as a ratio of protein levels with TMP treatment divided by Akt(WT) protein levels without TMP treatment. Graph shows means with SEM. N = 3 replicate samples per condition. ****p < 0.0001; n.s. vs. DD-Akt(WT)–TMP unless otherwise indicated, 2-way ANOVA with multiple comparisons.
(TIF)

S4 Fig Adding a second DD domain does not change inducibility or basal activity. HEK293 cells were transfected with constructs to overexpress single DD domain Akt(E40K) or double DD domain Akt(E40K) with varying linker combinations. Cells were treated with 10 μM TMP for 24 hr and then lysed for western blotting. Protein expression levels were quantified and normalized to ERK1 as a loading control. Fold induction was calculated as a ratio of protein levels with TMP treatment divided by protein levels without TMP treatment. Graph shows means with SEM. N = 2 independent experiments with 2–3 replicates per condition per experiment. *p < 0.05 vs. DD-Akt(E40K), n.s. determined through 2-way ANOVA with multiple comparisons.
(TIF)
