# Supplementary figures and images for: Context-dependent expression of a conditionally-inducible form of active Akt
Source: PLoS One. 2018 Jun 19;13(6):e0197899. doi: 10.1371/journal.pone.0197899 (PMC6007834; doi:10.1371/journal.pone.0197899)

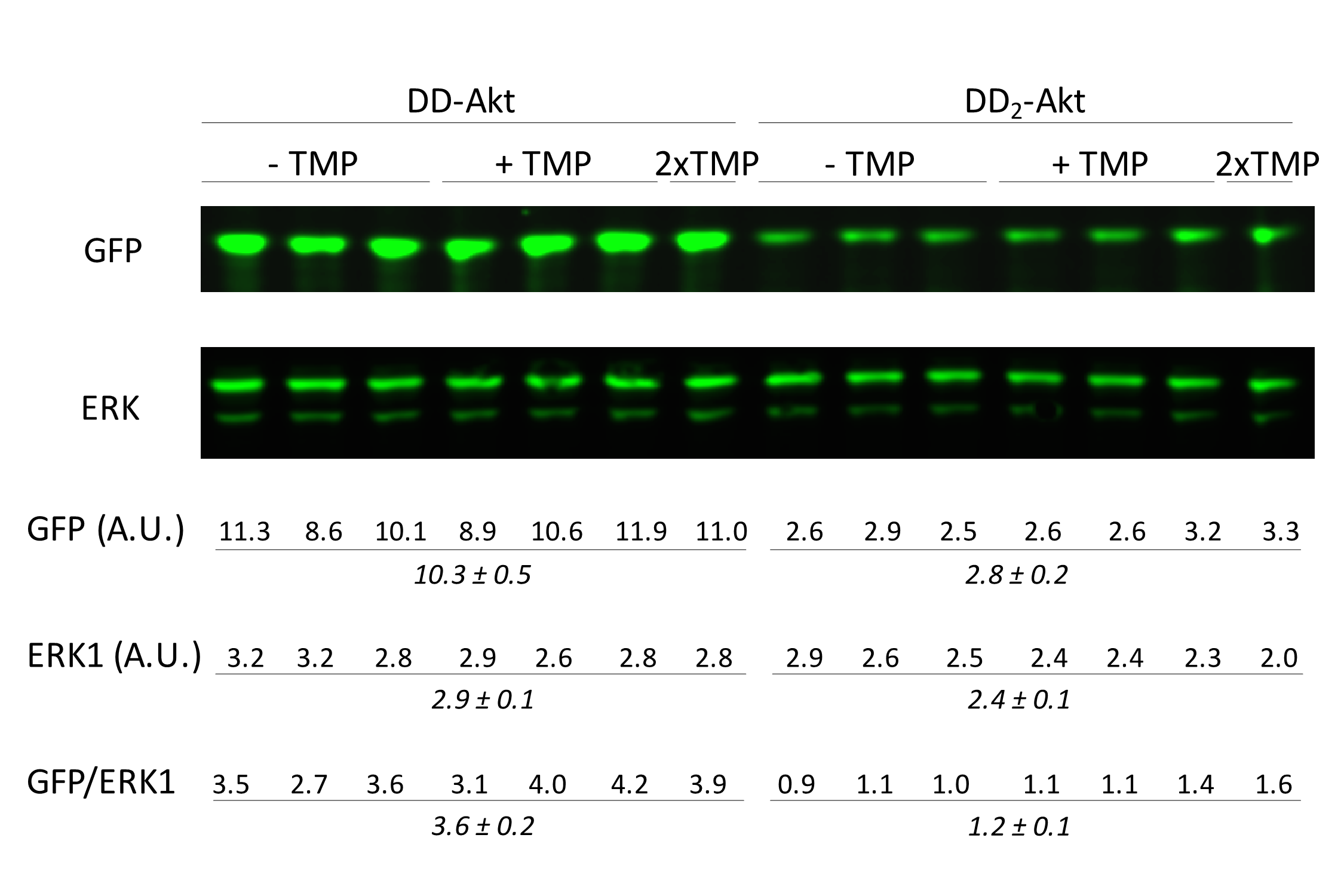

Supplement: S1 Fig — Upper image shows western blot of EGFP (GFP) and ERK loading controls for an experiment in which replicate cultures of PC12 cells were infected with lentivirus expressing either DD-Akt(E40K) or DD2-Akt(E40K) as indicated, and treated for 24 hr with or without TMP (10 μM) or 2xTMP (20 μM) and then lysed. The infection efficiencies were 78.3% and 75.6%, respectively as determined by immunofluorescence imaging for EGFP. Blots were probed for ERK and EGFP (GFP) proteins and relative intensities of the indicated bands were determined as described in Materials and Methods. Relative intensities are given in Arbitrary Units (A.U.) for each individual replicate for both GFP and ERK1 as well as the ratios thereof. Mean values for each transfection condition (DD-Akt or DD2-Akt) are given in italics ± S.E.M. and indicate consistency of transfection from replicate to replicate and of the GFP/ERK1 ratio. (TIF) [file pone.0197899.s001.tif]

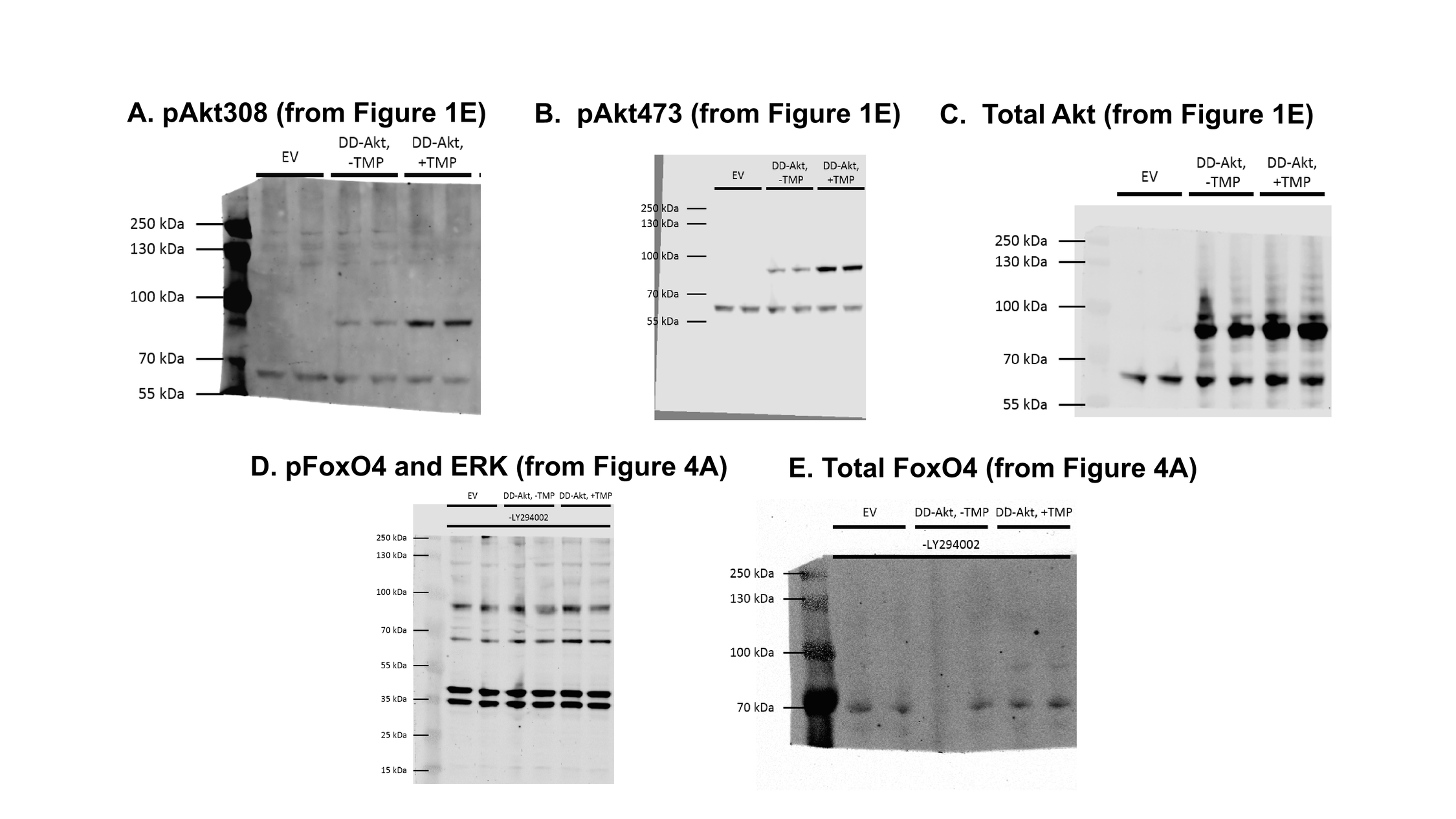

Supplement: S2 Fig — Uncropped blots with molecular weight markers for Fig 1E (panels A-C) and 4A (panels D,E). Blot for total Akt is more highly exposed than in Fig 1 to show background. In each panel 6 irrelevant lanes to the right are not included. (TIF) [file pone.0197899.s002.tif]

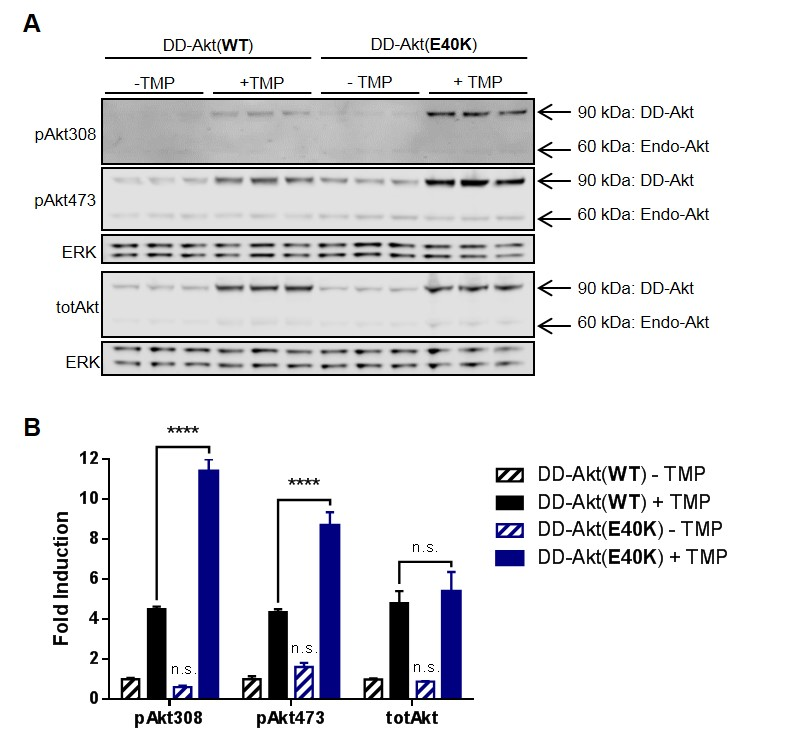

Supplement: S3 Fig — HEK293 cells were transfected with DD constructs with WT Akt or Akt(E40K). Cells were treated with 10 μM TMP for 24 hr and then lysed for western blotting. Protein expression levels were quantified and normalized to ERK1 as a loading control. Fold induction was calculated as a ratio of protein levels with TMP treatment divided by Akt(WT) protein levels without TMP treatment. Graph shows means with SEM. N = 3 replicate samples per condition. ****p < 0.0001; n.s. vs. DD-Akt(WT)–TMP unless otherwise indicated, 2-way ANOVA with multiple comparisons. (TIF) [file pone.0197899.s003.tif]

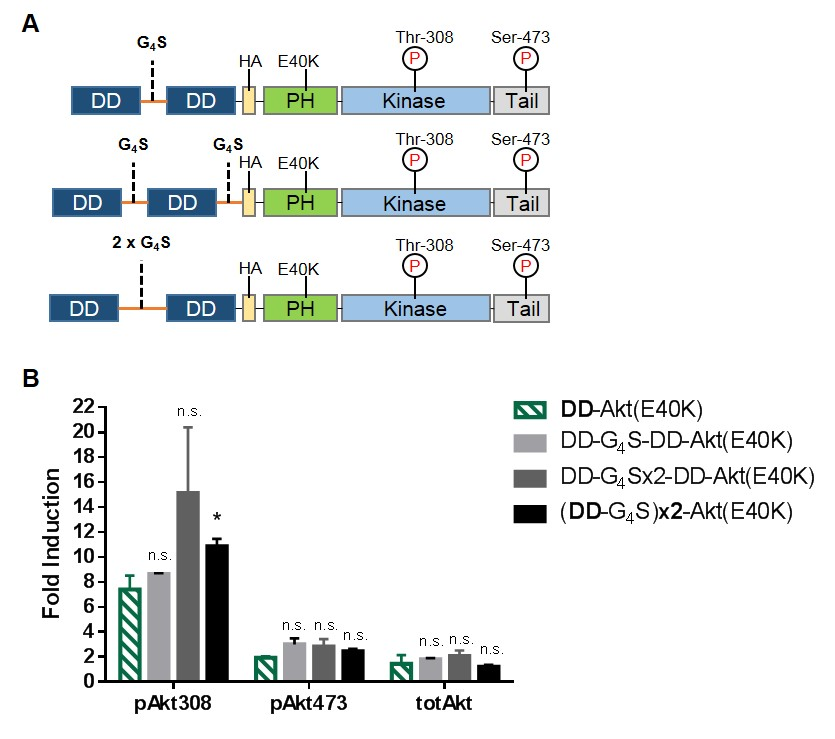

Supplement: S4 Fig — HEK293 cells were transfected with constructs to overexpress single DD domain Akt(E40K) or double DD domain Akt(E40K) with varying linker combinations. Cells were treated with 10 μM TMP for 24 hr and then lysed for western blotting. Protein expression levels were quantified and normalized to ERK1 as a loading control. Fold induction was calculated as a ratio of protein levels with TMP treatment divided by protein levels without TMP treatment. Graph shows means with SEM. N = 2 independent experiments with 2–3 replicates per condition per experiment. *p < 0.05 vs. DD-Akt(E40K), n.s. determined through 2-way ANOVA with multiple comparisons. (TIF) [file pone.0197899.s004.tif]
